# Clinical Utility of Mindfulness Training in the Treatment of Fatigue After Stroke, Traumatic Brain Injury and Multiple Sclerosis: A Systematic Literature Review and Meta-analysis

**DOI:** 10.3389/fpsyg.2016.00912

**Published:** 2016-06-23

**Authors:** Kristine M. Ulrichsen, Tobias Kaufmann, Erlend S. Dørum, Knut K. Kolskår, Geneviève Richard, Dag Alnæs, Tone J. Arneberg, Lars T. Westlye, Jan E. Nordvik

**Affiliations:** ^1^Sunnaas Rehabilitation Hospital HT, NesoddenNorway; ^2^KG Jebsen Centre for Psychosis Research, Division of Mental Health and Addiction, NORMENT: Norwegian Centre for Mental Disorders Research, Oslo University Hospital and Institute of Clinical Medicine, University of Oslo, OsloNorway; ^3^Department of Psychology, University of Oslo, OsloNorway; ^4^Department of Behavioural Sciences, Oslo and Akershus University College of Applied Sciences, OsloNorway

**Keywords:** fatigue, stroke, traumatic brain injury, multiple sclerosis, mindfulness, meta-analysis

## Abstract

**Background:** Fatigue is a common symptom following neurological illnesses and injuries, and is rated as one of the most debilitating sequela in conditions such as stroke, traumatic brain injury (TBI), and multiple sclerosis (MS). Yet effective treatments are lacking, suggesting a pressing need for a better understanding of its etiology and mechanisms that may alleviate the symptoms. Recently mindfulness-based interventions have demonstrated promising results for fatigue symptom relief.

**Objective:** Investigate the efficacy of mindfulness-based interventions for fatigue across neurological conditions and acquired brain injuries.

**Materials and Methods:** Systematic literature searches were conducted in *PubMed*, *Medline*, *Web of Science*, and *PsycINFO*. We included randomized controlled trials applying mindfulness-based interventions in patients with neurological conditions or acquired brain injuries. Four studies (*N* = 257) were retained for meta-analysis. The studies included patients diagnosed with MS, TBI, and stroke.

**Results:** The estimated effect size for the total sample was -0.37 (95% CI: -0.58, -0.17).

**Conclusion:** The results indicate that mindfulness-based interventions may relieve fatigue in neurological conditions such as stroke, TBI, and MS. However, the effect size is moderate, and further research is needed in order to determine the effect and improve our understanding of how mindfulness-based interventions affect fatigue symptom perception in patients with neurological conditions.

## Introduction

Fatigue is a prevalent condition associated with a number of diseases (Hofman et al., 2007; Kluger et al., 2013). Curative pharmacologic or non-pharmacologic treatments have not yet been identified (Lee et al., 2008; Cantor et al., 2014; Wu et al., 2015). In some cases, like cancer-related fatigue, mindfulness-based interventions have resulted in significant reduction of fatigue symptoms (Pachman et al., 2014). However, the mechanisms of fatigue are poorly understood (Kutlubaev et al., 2012; Wu et al., 2015). Neurological diseases cause harm to the central nervous system, and fatigue associated with these kinds of diagnoses may have another etiology and respond differently to treatment than other types of fatigue. The objective of this systematic review is to investigate the effect of mindfulness-based interventions on fatigue symptoms following neurological conditions and acquired brain injuries.

Fatigue and increased fatigability are reported in a range of neurological conditions, like Parkinson’s, traumatic brain injury (TBI), myasthenia gravis, stroke, and multiple sclerosis (MS; Chaudhuri and Behan, 2004; DeLuca, 2005; Colle et al., 2006; Barker-Collo et al., 2007; Cantor et al., 2008; Friedman et al., 2011; Kluger et al., 2013). Often manifested as a mental and physical lack of energy, increased tiredness and reduced initiative (Glader et al., 2002; Choi-Kwon and Kim, 2011), fatigue can be persistent (Duncan et al., 2012; Ponsford et al., 2014) and pose a serious barrier to rehabilitation (Michael, 2002). Moreover, fatigue is associated with negative outcomes such as lower levels of functioning (Juengst et al., 2013), reduced quality of life, increased institutionalization and mortality (Glader et al., 2002; Lerdal et al., 2009).

Self-reports confirm fatigue as a distressing condition, and it is rated as the worst or one of the worst symptoms by 55, 50, and 40% of MS, TBI, and stroke patients, respectively (Fisk et al., 1994; LaChapelle and Finlayson, 1998; Ingles et al., 1999). Adding to the impact of the symptoms, nearly half of the stroke patients suffering from fatigue felt that they were offered insufficient help managing the fatigue (McKevitt et al., 2010). Due to the debilitating consequences, and incomplete understanding of the mechanisms and treatments of fatigue, the topic was listed among the top ten research priorities in a consensus report from UK stroke survivors, caregivers, and health professionals (Pollock et al., 2014). Such reports are in line with the literature on fatigue in other neurological conditions, often emphasizing the substantial limitations in our comprehension and treatment of this symptom (Chaudhuri and Behan, 2004).

Agreeing on a universally accepted definition of fatigue has proven problematic (Chaudhuri and Behan, 2004; Immink, 2014). Although the search for biological correlates of fatigue is ongoing, the experience of fatigue is fundamentally subjective (Chaudhuri and Behan, 2004; Dittner et al., 2004), and common not only to ill health but also in draining physical activities. The feeling of fatigue is thus both intimate, yet universal, and the experience has proven difficult to quantify and measure objectively (Dittner et al., 2004; Belmont et al., 2006; Mollayeva et al., 2013). However, it is generally accepted to differentiate “normal” fatigue from “pathological” fatigue (de Groot et al., 2003). While normal fatigue is considered to reflect a state of weariness associated with strain that can be lessened by rest, serving a protective and restorative function (Choi-Kwon and Kim, 2011), the pathological fatigue seen in many patients with acquired brain injury and MS tends to be more persistent, less related to strain, abnormal, excessive, and problematic (de Groot et al., 2003).

Fatigue is frequently measured by self-report questionnaires (Belmont et al., 2006; Lerdal et al., 2009). A multitude of scales are available [see Krupp et al., 1989, Fatigue Severity Scale (FSS); Schwartz et al., 1993, Fatigue Assessment Instrument (FAI); Smets et al., 1995, Modified Fatigue Impact Scale (MFIS)], and the same tools are often used across conditions (Dittner et al., 2004). Besides self-report measures, performance-based tests are sometimes also applied (Lerdal et al., 2009), presumably measuring more objective aspects of fatigue, but no objective “gold standard” or litmus test is currently available (Dittner et al., 2004).

Why some people develop fatigue in the face of neurological injury or disease, whereas others do not, is yet to be answered. Several factors have been found to be associated with or predict post-stroke fatigue (PSF), including functional impairment severity, depression, pain, sleep disturbances, cognitive impairments, physical deconditioning, pre-stroke fatigue, sedative medications, coronary heart disease, and increasing age (Mead et al., 2011; Wang et al., 2014). The variety of predictors suggests a complex etiology, further complicating treatment and diagnostic assessments. As fatigue is a pivotal part of both depression and sleep disturbances, these conditions have been posed as alternative or supplementing explanations (Kos et al., 2007). There are, however, many patients experiencing fatigue without reporting depression (van der Werf et al., 2001; Glader et al., 2002), and some studies fail to find associations between fatigue and sleep problems (Schepers et al., 2006). Findings like this may indicate that fatigue can be a partly independent symptom, and neurological fatigue in the absence of conditions such as depression or sleep problems has been referred to as “primary fatigue” (Forwell et al., 2008).

Owing to the multidimensionality of fatigue, the construct is often conceptualized as reflecting different subcategories. An applicable categorization is the division between peripheral (predominantly physical/muscular) and brain-derived central (more psychologically rooted, the sense of complete exhaustion) fatigue (Chaudhuri and Behan, 2000, 2004), in which mental fatigue constitutes an important dimension of the latter (Chaudhuri and Behan, 2000, 2004). The coping hypothesis offers one explanation for this experience, stating that increased fatigue is rooted in the continuous effort needed in order to compensate for cognitive impairments caused by the brain injury (Van Zomeren et al., 1984). Indeed, a few studies have reported that individuals with TBI and MS are showing increased brain activation compared to healthy controls while performing cognitive tasks (McAllister et al., 1999, 2001; DeLuca et al., 2008; Kohl et al., 2009) possibly indicating increased mental effort, while yet other studies have identified reduced sustained attention (McAvinue et al., 2005), selective attention deficits (Ziino and Ponsford, 2006), and a tendency to stimulus over-selectivity (McHugh and Wood, 2013) in patients with TBI. These findings are in line with the hypothesis of a central, brain-derived fatigue in neurological conditions, which might be associated with disruptions in circuits involving basal ganglia, frontal cortex thalamus (Chaudhuri and Behan, 2000). As pointed out by Chaudhuri and Behan (2004), (central) fatigue is “consistently seen with lesions in pathways associated with arousal and attention, reticular and limbic systems and basal ganglia” (pp. 979–980).

The models, hypotheses and correlates reviewed above are by no means offering an exhaustive account of the mechanisms of fatigue in neurological conditions. Still, by highlighting relevant aspects of the condition, such accounts may provide clues about potentially effective treatment strategies. Attentional impairments seem to be a recurrent and relevant aspect. Thus, in order to improve attentional regulation and control, a treatment procedure that has been suggested, among others, is mindfulness-based training (Chen et al., 2011; McHugh and Wood, 2013).

### The Potential of Mindfulness-Based Interventions

Mindfulness-based approaches are frequently associated with psychological health and wellbeing (Keng et al., 2011). It can be considered as both a process and an outcome; through regular mindful practice (process) one seeks to cultivate a mindful awareness (outcome) (Shapiro and Carlson, 2009). The latter has been described as a non-judgmental, open and intentional awareness of the present and constantly unfolding experience (Kabat-Zinn, 2003), thus reflecting the antithesis of rumination and avoidance. According to Kang et al. (2013), mindfulness-based stress reduction (MBSR) is thought to contribute to health and well-being by reducing the impact of maladaptive coping strategies and fixed, reflexive behavioral, emotional and cognitive patterns.

Originating from ancient Eastern philosophy and Buddhism, mindfulness-based interventions are now frequently applied within the context of the modern health care systems, and a growing body of literature indicates its potential as a beneficial intervention (Mars and Abbey, 2010). A meta-analysis on MBSR comprising 20 studies and 1605 participants, provided support for its utility in a wide range of clinical populations (Grossman et al., 2004). More specifically, several studies indicate that mindfulness-based interventions have positive effects on anxiety and depression (Evans et al., 2008; Hofmann et al., 2010), and mindfulness-based interventions have been associated with reduced fatigue in patients with cancer (Carlson and Garland, 2005; van der Lee and Garssen, 2012) and chronic fatigue syndrome (Surawy et al., 2005).

### Mindfulness and Fatigue in Neurological Conditions: Rationale for a Review

Although fatigue is a common and debilitating symptom associated with a range of neurological conditions, the mechanisms are poorly understood, and our knowledge about potentially effective treatment strategies is limited (Chaudhuri and Behan, 2004; Kluger et al., 2013). Encouragingly, mindfulness-based approaches have demonstrated positive effects on both general health and fatigue in other, non-neurological conditions (Grossman et al., 2004; Carlson and Garland, 2005; van der Lee and Garssen, 2012). Moreover, emerging evidence suggests associations between mindfulness meditation and certain aspects of attentional functioning (Chambers et al., 2008; Moore and Malinowski, 2009) and it has been hypothesized that the positive effects of mindfulness in neurological conditions are associated with improved self-regulation (Immink, 2014) and attentional processes (Valentine and Sweet, 1999). Indeed, since non-elaborative observation of the moment-to-moment experience requires the ability to anchor attention, as well as an ability to shift the attention between different aspects of experience (Keng et al., 2011), self-regulation of attentional processes is vital to mindfulness. Studies have identified both neurobiological correlates, i.e., increased gray matter density (Pickut et al., 2013) and alterations in brain connectivity (Kilpatrick et al., 2011) associated with mindfulness training. As described above, attentional disturbances have been implicated in fatigue in TBI and stroke (Chaudhuri and Behan, 2000, 2004), which may make mindfulness-based strategies particularly beneficial for these patients.

Whereas previous reviews summarizing the literature on mindfulness and fatigue in neurological disorders (Immink, 2014), stroke (Lawrence et al., 2013), and MS (Simpson et al., 2014) have been published, none of the mentioned have conducted a meta-analysis of the treatment effects. Moreover, as many of the relevant trials have been conducted in small samples, pooling results in a meta-analysis will be particularly useful with regards to improving accuracy in effect estimates (Walker et al., 2008).

Consequently, our main aim is to present an updated systematic review and a meta-analysis of mindfulness-based treatment of fatigue in neurological conditions and acquired brain injuries. Due to the sparsity of disease-specific treatment studies, considering fatigue across different neurological diagnoses in one review is considered expedient at this early stage of research, where the emphasis is on exploring whether mindfulness-based treatment should be considered a relevant intervention in neurological conditioned fatigue. Moreover, fatigue in neurological conditions (such as, i.e., TBI, stroke, and MS) share several critical features with regards to both experience and consequences (Mills et al., 2012; Eilertsen et al., 2015) and it has previously been suggested that fatigue in neurological conditions may respond to the same treatments (de Groot et al., 2003).

## Materials and Methods

### Database Searches

Searches were conducted in PubMed (from 1966), PsycINFO (from 1806), and Web of Science including Medline on May 16, 2016. Applied keywords were “fatigue” and “mindfulness.”

### Criteria for Inclusion in This Review

Randomized and quasi-randomized controlled trials aiming to measure the effect of different interventions on fatigue associated with neurological conditions and acquired brain injuries were included. We included studies who were primarily targeting fatigue, and studies who included fatigue as a secondary outcome measure.

Various kinds of controlled trials were included. Studies had to have some kind of control arm, but we did include studies with both active (i.e., another form of treatment) and passive (e.g., wait list control) control conditions. We did not differentiate between blinded and non-blinded procedures. In order to be included in the present review, studies had to report fatigue symptoms as continuous variables measured on a fatigue scale. Studies also had to report at least mean and standard deviations (SDs) on the relevant fatigue scale pre- and post-treatment per group, which is required for a meta-analysis. Alternatively, in instances where this criterion was not fulfilled, we contacted authors with a request to provide the necessary data in order to be included. Only published, peer-reviewed studies were included. We restricted the search to publications written in English, Norwegian, Swedish, and Danish.

### Exclusion Criteria

We did not include trials registering fatigue only as a potential side effect of treatment, or as a contraindication for treatment (usually feasibility studies), or studies targeting parallel, but different conditions to fatigue, such as sleepiness, reduced vigilance, anxiety, and depression.

### Study Participants

Only studies in which participants were aged 18 years or older were included in this review. Participants did not need to exhibit fatigue at recruitment. All participants had been diagnosed with a neurological illness or an acquired injury to the central nervous system, but we did not apply any criterions regarding time since injury/debut of illness. All subtypes of stroke were included. In line with Parikh et al. (2007), we defined TBI as “physical injury to brain tissue that temporarily or permanently impairs brain function” (p. 119).

### Study Interventions

Only group-oriented, mindfulness-based interventions were included. To avoid extensive heterogeneity in interventions, we excluded interventions based primarily on physical training and/or mindfulness in *movement*, i.e., Tai Chi, Qi Gong, and yoga.

### Study Measures

We included all continuous self-report measures of fatigue.

### Selection of Studies

Articles were identified and duplicates were removed. We used Rayyan, a systematic reviews web app, for exploring and filtering searches for eligible studies (Elmagarmid et al., 2014). The remaining publications were further screened for relevance by two independent authors (JN and KU). This was done by reading all titles and abstracts, retaining studies that (i) applied a mindfulness-based intervention, (ii) mentioned fatigue as an outcome, and (iii) included patients with neurological illnesses or acquired brain injuries. Full texts were then obtained for all the potentially relevant studies, and these were individually scrutinized according to the inclusion criteria by both JN and KU. Whenever conflicting evaluations arose, or when the assessors were unsure whether to include a study or not, co-authors LW and TK were consulted. Studies failing to meet the inclusion criteria were excluded.

### Data Extraction

In order to extract and organize data, all relevant information was collected in a data-extraction form based on a schema from Cochrane, modified to fit the needs of this current review. Relevant study information included details on design, control, and treatment conditions, the main objective, participant characteristics (whether or not they had fatigue at recruitment, mean age, sex, and time since injury plus type of injury), sample size, fatigue measures and associated scores (SDs and means if available), type of main analysis, and main conclusion.

### Data Synthesis

We performed the meta-analysis-based on standardized mean changes between pre- and post-intervention (Becker, 1988). First, we extracted the necessary values for mean and SD pre- and post-intervention for treatment and control groups from the publications. In cases where these values were not directly accessible, we computed post-means from change scores. Second, if there were several treatment groups included, we combined them into one treatment group, following the procedures described in Cochrane (Higgins and Green, 2011, Table 7.7a). Treatment groups comprising participants that served as the control group initially and that were offered treatment afterward were not considered. Third, for each study and group, we estimated pretest–posttest correlation coefficients. If raw data was available, these were computed using Pearson correlation, whereas we assumed a coefficient of *r* = 0.7 for the other studies. The coefficient of *r* = 0.7 was based on the mean of the available correlations (mean *r* = 0.67). Finally, we performed the meta-analysis using the metafor package in R (Viechtbauer, 2010). Briefly, we computed the difference in standardized mean change for each group and implemented a fixed-effects model to estimate effect size across studies. Statistical heterogeneity was assessed using a *Q* test. In addition, to rule out any bias due to a wrongly chosen pretest–posttest coefficient, we reran the analysis 21 times, simulating coefficients of the full range from *r* = -1–1 in steps of 0.1. Finally, since the procedure implemented in the metafor package does not allow accounting for subject drop out from pre- to post-intervention, we reran the analysis twice, once using the sample sizes from pre-intervention and once using the sample sizes from post-intervention. The main results reported below were based on the smaller sample sizes from post-intervention.

## Results

### Literature Search

**FIGURE 1:**
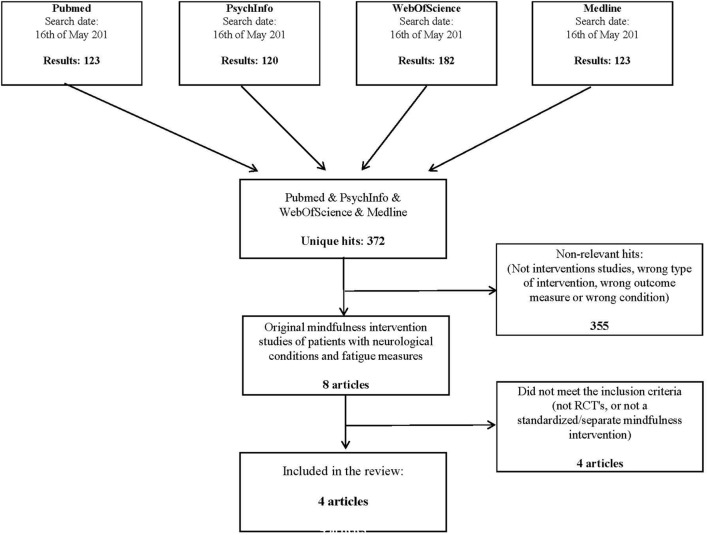
**Flowchart showing the search process and the selection of studies**.

The search yielded 372 unique articles referring to fatigue and mindfulness. Based on the screening and selection process described in the Section “Materials and Methods,” four papers were found to satisfy the inclusion criteria and were retained for analysis (see flow chart, **Figure 1**).

The included studies are displayed in **Table 1**. Excluded studies are listed in **Table 2** along with the reasons for exclusion.

**Table 1:** Study characteristics.

Author (country)	Participant details	Type of intervention; duration	Control group	Outcome measures (relevant to fatigue)	Data collection
Grossman et al., 2010 (Switzerland)	*N* = 150 Attrition: 5% Condition: MS Fatigue as inclusion criterion? No	Closely followed the MBSR-program, included goal definition, weekly mindfulness classes, homework. Duration 8 weeks.	Usual care	MFIS (Modified Fatigue Impact Scale)	Baseline Post-intervention 6 months post-intervention
Johansson et al., 2012 (Sweden)	*N* = 29 Attrition: 3 subjects Condition: 12 stroke, 10 TBI Fatigue as inclusion criterion? Yes	Based on MBSR. Included yoga, body scan and sitting mediation, home practice. Duration 8 weeks.	Waiting-list	MFS (Self-assessment Mental Fatigue Scale)	Baseline Post-intervention (+ post-intervention for CG after attending MBSR)
Johansson et al., 2015 (Sweden)	*N* = 38 Attrition: 4 subjects Condition: 18 stroke, 16 TBI Fatigue as inclusion criterion? Yes	Two independent intervention groups: MBSR face-to-face, MBSR (interactive) delivered on the internet. Duration 8 weeks.	Group walks in garden	MFS (Self-assessment Mental Fatigue Scale)	Baseline Post-intervention (+ post-intervention for CG after attending MBSR)
Bogosian et al., 2015 (UK)	*N* = 40 Attrition: 5 subjects Condition: MS Fatigue as inclusion criterion? No	Based on MBCT, delivered via Skype group-based video-conferences. Included mindfulness practice and homework. Duration 8 weeks.	Waiting-list	FSS (Fatigue Severity Scale)	Baseline Post-intervention 3 months post-intervention

*N = *N* before drop out; CG = control group.*

**Table 2:** Excluded studies.

Study	Reason for exclusion
Hofer et al., 2014	The study was a single-arm trial that tested a program for treating PSF.
Mackay et al., 2015	Mindfulness included only as a smaller part of an integrative intervention (relaxation, mindfulness, social support, and education). Two active treatment groups—mindfulness in both groups.
Johansson et al., 2015	14 of the 22 participants had completed a previous MBSR-program. The study based on this (previous) MBSR-program is already included in this review (see Johansson et al., 2012).
Burschka et al., 2014	Although including some elements with resemblance to mindfulness training, the intervention was based on Tai Chi and Tai Chi training.

Among the four included studies (257 participants all together), two were primarily targeting (mental) fatigue (Johansson et al., 2012, 2015; a total of 63 participants), while the other two studies (with a total of 193 participants) reported fatigue as either a secondary outcome measure (Bogosian et al., 2015) or as one out of several main outcome measures (Grossman et al., 2004). In the studies specifically aimed at alleviating fatigue, all participants reported fatigue at recruitment, whereas this was not always the case in the remaining two studies. In two of the studies (Johansson et al., 2012, 2015), all participants had either stroke or TBI (*n* = 63, stroke and TBI combined). In the studies from Grossman et al. (2004) and Bogosian et al. (2015), all participants were diagnosed with MS. All interventions were based on either MBSR (Grossman et al., 2004; Johansson et al., 2012, 2015) or mindfulness-based cognitive therapy (MBCT; Bogosian et al., 2015), and all interventions lasted 8 weeks.

### Fatigue Questionnaires

Both studies of Johansson and colleagues (Johansson et al., 2012, 2015) used a questionnaire of mental fatigue (Mental Fatigue Scale, MFS; Johansson et al., 2010). Bogosian et al. (2015) applied the FSS (Krupp et al., 1989), while Grossman et al. (2004) measured fatigue by the MFIS, based on Fatigue Impact Scale (Fisk et al., 1994). Both MFIS and FSS have been classified as valid fatigue measures in MS (Flachenecker et al., 2002). All of the included measures were based on self-report. They were all continuous, indicating increased fatigue severity by increased score on the scale.

### Risk of Bias/Validity Assessment

Only Grossman et al. (2004) and Bogosian et al. (2015) explicitly reported convincing procedures of randomization and allocation by concealment. In spite of randomization procedures, the groups in Bogosian et al.’s (2015) study differed substantially on the fatigue measure pretreatment (FSS-scores MBCT-group: 39.9; control group: 48.2). This is not discussed in their report, although Bogosian did make a note of this in a personal e-mail communication with us. Grossman et al. (2004) found no significant between groups differences at baseline, but the fatigue variable (MFIS) differed some (*p* = 0.06, intervention group: 35.15; control group: 30.28). Analyses of covariance with mean adjusted for pre-intervention level were, therefore, reported on the fatigue-variable. Johansson et al. (2012) also randomized allocation of participants, but the report did not specify the specific randomization procedure.

Aiming to ensure equal groups pre-intervention, Johansson et al. (2012, 2015) all investigated group differences. In Johansson et al. (2012), time since injury was found to be significantly longer in the intervention group. However, time since injury was not significantly correlated to other variables. In Johansson et al. (2015), no systematic group differences prior to treatment were identified. Further, intention-to-treat analyses were conducted with only the randomized participants to control for the insufficient randomization.

All four studies reported attrition rate and reasons for drop out. Attrition rates were generally low, with the highest rate being about 12%, or 4 out of 34 (Johansson et al., 2015) while Grossman et al. (2010) reported the lowest drop out with 5% in the intervention group.

### Meta Analysis Results

**FIGURE 2:**
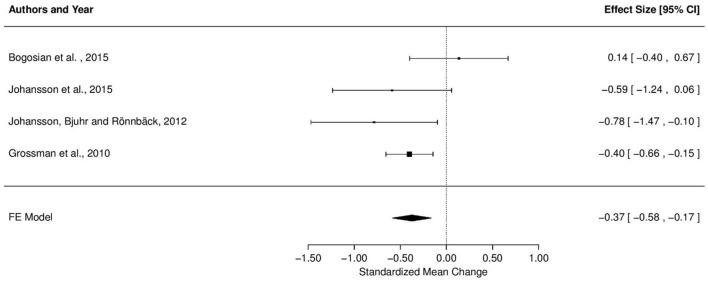
**Effect of treatment on fatigue severity, comparison of effect sizes**.

**Figure 2** shows a forest plot comparing effect sizes between the four studies and the resulting pooled estimate from fixed effect modeling across studies. We estimated a significant effect of treatment across studies (effect size: -0.37, CI: -0.58 to -0.17, *p* < 0.01).

The *Q* test for statistical heterogeneity was not significant [*Q*(3) = 5.33, *p* = 0.15]. Direction of effect was unaffected by the simulated pretest–posttest correlation coefficients, with effect sizes varying from -0.28 to -0.42 when coefficients were varied from *r* = -1 to 1. Importantly, the model was significant for all realistic (positive) estimates of *r*. Further, the effect size was unaffected by the sample sizes used to compute the standardized mean change (pre- or post-sample size, both effect sizes -0.37).

## Discussion

By the use of systematic literature search and meta-analysis, our aim was to evaluate the effect of mindfulness-based interventions in alleviating fatigue associated with neurological conditions and acquired brain injuries. We identified four relevant studies, targeting fatigue in stroke, TBI, and MS. Based on the four studies (*N* = 257) included in the final analysis, we estimated the total effect of mindfulness-based interventions on fatigue scores with an effect size of -0.37 across studies. These results indicate a positive effect of mindfulness intervention on treating fatigue compared to no treatment or control treatments.

Our estimated effect size of 0.37 for the treatment effect of mindfulness-based interventions in stroke, TBI, and MS matches well with the results from meta-analyses of the treatment effect of mindfulness-based interventions on other clinical conditions. MBSR’s effect on mental health for people with cancer was estimated with an effect size of 0.35 (Ledesma and Kumano, 2009) and MBSR’s treatment effect on psychological distress for adults with various chronic medical diseases was estimated with an effect size of 0.32 (Bohlmeijer et al., 2010). Further, a meta-analysis by Grossman et al. (2004) estimated an even stronger effect of 0.54 for MBSR’s effect on mental health variables in a sample comprising both clinical populations and non-clinical populations. While differences in strength of the estimated treatment effect is likely explained by a variety of factors such as different populations, sample sizes, diagnosis, inclusion criteria, and statistical choices across studies, a common feature of all meta-analyses is nonetheless a significant positive effect of mindfulness-based treatment.

Similar differences may explain the substantial variation of reported effects across the four studies included in this meta-analysis. As depicted in **Figure 1**, three of the studies yielded a positive treatment effect, whereas one study (Bogosian et al., 2015) reported a slightly negative effect of MBSR intervention. However, as the latter effect is small (0.14) with confidence intervals ranging from positive and negative treatment effects, these results should not be treated as an indication of a harmful treatment effect.

The strongest effects of treatment were seen in the two studies from Johansson and colleagues (-0.59 and -0.78; Johansson et al., 2012, 2015). These studies were also the only two studies requiring above-cutoff fatigue at recruitment. Thus, the strong effect sizes may well reflect a stronger benefit from treatment for more severely fatigued patients. Differences in the assessment of fatigue may, however, provide an alternative explanation for the strength of effect. Johansson and colleagues were measuring mental fatigue by the MFS, whereas the remaining studies applied measures of more general subjective fatigue, namely the FSS and MFIS. This difference may thus alternatively suggest higher effectiveness of mindfulness-based interventions in alleviating mental fatigue specifically compared to treating fatigue in general.

It should be noted that although Johansson et al. (2015) employed patient randomization when possible, seven participants were allowed to choose intervention group based on personal preferences, and some participants were placed in the Internet group due to geographical considerations. When including participants that were allowed to choose which intervention group to follow (Johansson et al., 2012) there is a risk of a selection bias. Whereas this would compromise the possibility to make valid comparisons between the two types of interventions (traditional, face-to-face MBSR versus Internet-based MBSR), the potential selection effects are not considered a major drawback for this current meta-analysis. This is mainly due to the fact that we are not addressing the question of whether MBSR is most effective when administered face-to-face or via Internet, but rather *if*, and to which extent, mindfulness-based interventions have an effect on neurological conditioned fatigue in general.

Another relevant issue is the question of how these interventions reduce fatigue. Through which mechanisms do the mindfulness training exert its positive effects? At this point we are not able to pin down the processes underlying symptom relief, and it is therefore still unclear whether any effect of mindfulness is related to the specific conditions included in this review or to a more general effect on well-being, further translated to an effect on fatigue. However, with reference to the hypothesis outlined in the Section “Introduction,” it seems plausible that mindfulness may be associated with improvements in self-regulation and attentional processes (Valentine and Sweet, 1999; Immink, 2014), counteracting fatigue through strengthening these functions. More indirect effects are also likely to be involved. Based on the assumption that mindfulness offers relief to several conditions, including depression and stress (Hofmann et al., 2010; Chiesa and Serretti, 2011; Garland et al., 2011), it is reasonable to assume that the observed reduction in fatigue originates partly from a reduction in comorbid stress and depression.

In the quest of identifying and detangling the mechanisms of intervention effect, research could benefit from looking to clinical treatment research and neuroscientific research combined. This interdisciplinary field, referred to as mindfulness neuroscience (Tang and Posner, 2013) are discovering relevant associations between mindfulness practice, brain networks and neuroplasticity, such as increased gray matter density (Pickut et al., 2013) and alterations in brain connectivity (Kilpatrick et al., 2011). Applying this approach specifically on fatigue in neurological conditions will perhaps contribute to a better understanding of the neurobiological mechanisms of fatigue, as well as whether and how, mindfulness can alleviate fatigue.

### Limitations

As for any meta-analysis, a few limitations should be taken into consideration. Above all, the number of studies in this meta-analysis is relatively low and statistical power may thus be limited, urging caution when drawing conclusions. The low power is a direct and unfortunate consequence of the lack of studies on this topic, rather than restrictive criteria for inclusion. We do, however, consider this small-scale analysis to make a relevant contribution to the literature insofar it identifies and draws attention to an area in need of clinical research, and prepares the ground for future research on fatigue by addressing a relevant line of treatment.

In addition, this analysis was performed across several conditions. While this may complicate the ability to detangle effects of treatment on the particular pathophysiology, combining stroke, TBI, and MS in one analysis renders feasible in this early stage of research. Firstly, stroke, TBI, and MS represent three major neurological conditions in which fatigue plays a pivotal role in relation to quality of life and level of functioning (Glader et al., 2002; Braley and Chervin, 2010; Juengst et al., 2013; Kluger et al., 2013). The fact that considerable levels of fatigue remain after controlling for other conditions and related comorbidities such as depression, suggests that fatigue is, at least partly, related to neurological disturbances in these conditions (Kluger et al., 2013). Further, fatigue in TBI, stroke, and MS share several critical features with regards to both experience and consequences (Mills et al., 2012; Eilertsen et al., 2015), and it has previously been suggested that fatigue in neurological conditions may respond to the same treatments (de Groot et al., 2003). However, patients suffering from TBI, stroke, and MS are likely to differ in age, prescribed medications and lifestyle, which may add heterogeneity to the current results. These differences would be interesting to address in future research.

Apart from limitations resulting from the inclusion of heterogeneous patient groups, the heterogeneity of procedures within and across the four studies comprises additional limitations. First, initial levels of fatigue showed substantial variation since not all included studies required participants to report fatigue upon recruitment. Second, heterogeneity was present in the conducted measures, as symptoms were reported along three different scales, as well as in the intervention procedures, as interventions were administered both face-to-face and online. However, the lack of standards for fatigue assessment poses a challenge in most research targeting fatigue (Immink, 2014). While application of online classes may well have some disadvantages compared with real life classes, the actual interventions delivered through web solutions have the strong advantage that they are standardized for all participants and correspond to the live versions of MBCT and MBSR, respectively.

In addition to these limitations based on differences in study procedures, we cannot exclude the possibility that uncontrolled factors exerted non-random effects on the results. For example, adverse effects of treatment were not targeted in this review. However, the generally low attrition rates give reason to believe that the majority of participants did not experience severe negative bi-effects of treatment. The presence of systematic differences in other relevant process variables such as individual expectations of symptom relief (Ledesma and Kumano, 2009), placebo effects or the feeling that therapy is not working, might also have influenced the results. And because not all studies included active control groups, the influence of placebo effects is impossible to evaluate accurately.

Finally, on a more general note, analyses based on pooled results from published studies will always be vulnerable to systematic biases like the file-drawer publication bias, suggesting that positive results are published in a greater extent than their non-significant or negative counterparts (Rosenthal, 1979; Felson, 1992). Whereas several methods for assessing publication bias in meta-analyses (e.g., the rank correlation test or regression to detect funnel plot asymmetry) are available, the power of such methods would be limited due to the low number of studies included in the current analysis (Sterne et al., 2000). This should urge us to treat results with caution. Moreover, because the search was restricted to publications written in English, Norwegian, Swedish, or Danish, we cannot rule out the possibility that relevant papers were missed due to language constraints.

## Conclusion

Our results suggest that mindfulness-based interventions have a potential to relieve fatigue in neurological conditions such as stroke, TBI, and MS. These results are in line with reviews in other domains, indicating positive effects of mindfulness-based treatments (Grossman et al., 2004; Ledesma and Kumano, 2009; Bohlmeijer et al., 2010). Still, the effect is modest, and future research needs to justify the strength and potential of mindfulness-based interventions in the treatment of fatigue, as to date only few studies with relatively low statistical power have assessed this intervention in neurological conditioned fatigue.

## Author Contributions

KU (First author): Leading the work with the systematic review and the development of the manuscript. Involved in all parts of the process, from idea to complete manuscript. TK (second author): Overlooking and interpreting the meta-analysis, and feed-back on all versions of the manuscript. ED: Feed back on the systematic review, the meta-analysis and the manuscript. KK: Feed back on the systematic review, the meta-analysis and the manuscript. GR: Feed back on the systematic review, the meta-analysis and the manuscript. DA: Interpretation of meta-analysis. Feed back on the systematic review, the meta-analysis and the manuscript. TA: Clinical psychologist with expertise in mindfulness. Feed back on the systematic review, the meta-analysis and the manuscript. LW (Senior author and corresponding author): Senior researcher involved in the development of the manuscript from idea to complete manuscript. Feed back on the systematic review, the meta-analysis and the manuscript. JN: Senior researcher involved in the development of the manuscript from idea to complete manuscript. Participation in the systematic review and providing the meta-analysis and the manuscript.

## Conflict of Interest Statement

The authors declare that the research was conducted in the absence of any commercial or financial relationships that could be construed as a potential conflict of interest.
